# Magnesium depletion score and depression: a positive correlation among US adults

**DOI:** 10.3389/fpubh.2024.1486434

**Published:** 2024-11-05

**Authors:** Wei Zhao, Hai Jin

**Affiliations:** ^1^Department of Neurology, Shanghai Putuo People’s Hospital, Tongji University, Shanghai, China; ^2^Department of Thoracic Surgery, Changhai Hospital, Naval Medical University, Shanghai, China

**Keywords:** depression, magnesium depletion score, micronutrients, magnesium, NHANES

## Abstract

**Background:**

The Magnesium depletion score (MDS) serves as a novel metric for quantifying magnesium deficiency in the human body, comprehensively assessing four indicators: diuretic use, proton pump inhibitor use, estimated glomerular filtration rate, and alcohol abuse. However, there have been no studies examining the potential association between MDS and depression.

**Methods:**

The study population for this cross-sectional study comprised adults from the National Health and Nutrition Examination Survey database from 2009 to 2018. Participants with a score of 10 or above on the Patient Health Questionnaire-9 were defined as having depression. We employed multivariable logistic regression models to investigate the association between MDS and depression. Furthermore, subgroup analyses were conducted to assess potential differences in this association among populations with diverse characteristics.

**Results:**

A total of 13,197 participants were included in this study. After adjusting for all covariates, a significant positive correlation was observed between MDS and depression. Specifically, for every unit increase in MDS, the likelihood of developing depression increased by 13% (OR = 1.13, 95% CI: 1.04–1.22, *p* = 0.0025). This positive correlation was consistent across MDS groups, with a 19% increase in depression likelihood in the medium group (OR = 1.19, 95% CI: 1.01–1.41, *p* = 0.0404) and a 58% increase in the high group (OR = 1.58, 95% CI: 1.21–2.07, *p* = 0.0007), using the low subgroup as a reference. Subgroup analyses revealed significant differences in the relationship between MDS and depression across races, marital statuses, and hypertension status.

**Conclusion:**

Our study has uncovered a significant positive association between MDS and depression. Reducing MDS in individuals may play a positive role in both the prevention and treatment of depression.

## 1 Introduction

Depression is a common mental disorder that has become one of the most important public problems worldwide. People with depression often exhibit negative self-esteem, low energy, loss of self-confidence, and even a high risk of suicide (1). The negative effects of depression are particularly severe in adults (2). According to the World Health Organization (3), 5 percent of adults globally suffer from depression. In 2020, depression adversely affected 17.2 percent of young adults aged 18 to 25 years in the United States, and there is a trend of continued growth in prevalence (4). This suggests that prevention and intervention for depression in adults is critical. However, there are still numerous challenges in the current treatment methods for depression, such as drug side effects and poor treatment adherence. Therefore, it is particularly important to search for safe and effective non-pharmacological treatment methods. In recent years, the role of micronutrients in depression has attracted attention. Numerous studies have demonstrated that some micronutrients affect depression through biological mechanisms, such as magnesium, zinc, and selenium (5–7).

Magnesium is one of the essential micronutrients that play a crucial role in human health and disease prevention (8). Its role in stress coping, neurotransmitter regulation, and other functions is irreplaceable (9). Previous studies have demonstrated that magnesium enhances neurocognitive functions and exhibits significant potential in the treatment of depression, suicidal behavior, and anxiety (10–12). Magnesium, as a safe and easily accessible nutrient, may emerge as a novel and promising treatment for depression. In research related to magnesium and depression, investigators frequently focus on magnesium levels or magnesium acquisition, encompassing serum magnesium levels and dietary magnesium intake (13, 14). However, it has been observed that serum magnesium levels and dietary magnesium intake do not accurately reflect the body’s magnesium content or its deficiency (15, 16). Therefore, there is an urgent need for an accurate, simple, and convenient tool that can be widely used to assess magnesium bioavailability.

In 2021, Fan et al. developed the Magnesium Depletion Score (MDS) (17) to assess the magnesium depletion status of the human body. The MDS takes into account four risk factors affecting the efficiency of renal magnesium absorption and is effective in identifying the degree of whole-body magnesium deficiency in humans. The MDS is more accurate and reliable than other magnesium-related clinical indicators (18). Since the MDS was proposed, many studies have confirmed its significant association with a variety of public health problems, such as hypertension, abdominal aortic calcification, and metabolic syndrome (19–21). However, to the best of our knowledge, no study has yet demonstrated whether there is an association between MDS and depression. To fill the gap in this research area, we provide new scientific evidence for the role of magnesium in the prevention and treatment of depression by deeply exploring the association between MDS and depression. Therefore, we used a cross-sectional study to verify whether MDS is associated with depression in US adults, utilizing data from the 2009–2018 National Health and Nutrition Examination Survey (NHANES).

## 2 Materials and methods

### 2.1 Inclusion of the population

In this study, all participants were selected from the NHANES database, a nationally representative survey administered by the National Center for Health Statistics (NCHS), a division of the Centers for Disease Control and Prevention. Before data collection, the NHANES database underwent a stringent ethical review and obtained approval from the NCHS Ethics Review Board. Moreover, all individuals involved in the study provided written informed consent. Interested parties can visit the official website for comprehensive details and updated information on the NHANES database.

**Figure 1:**
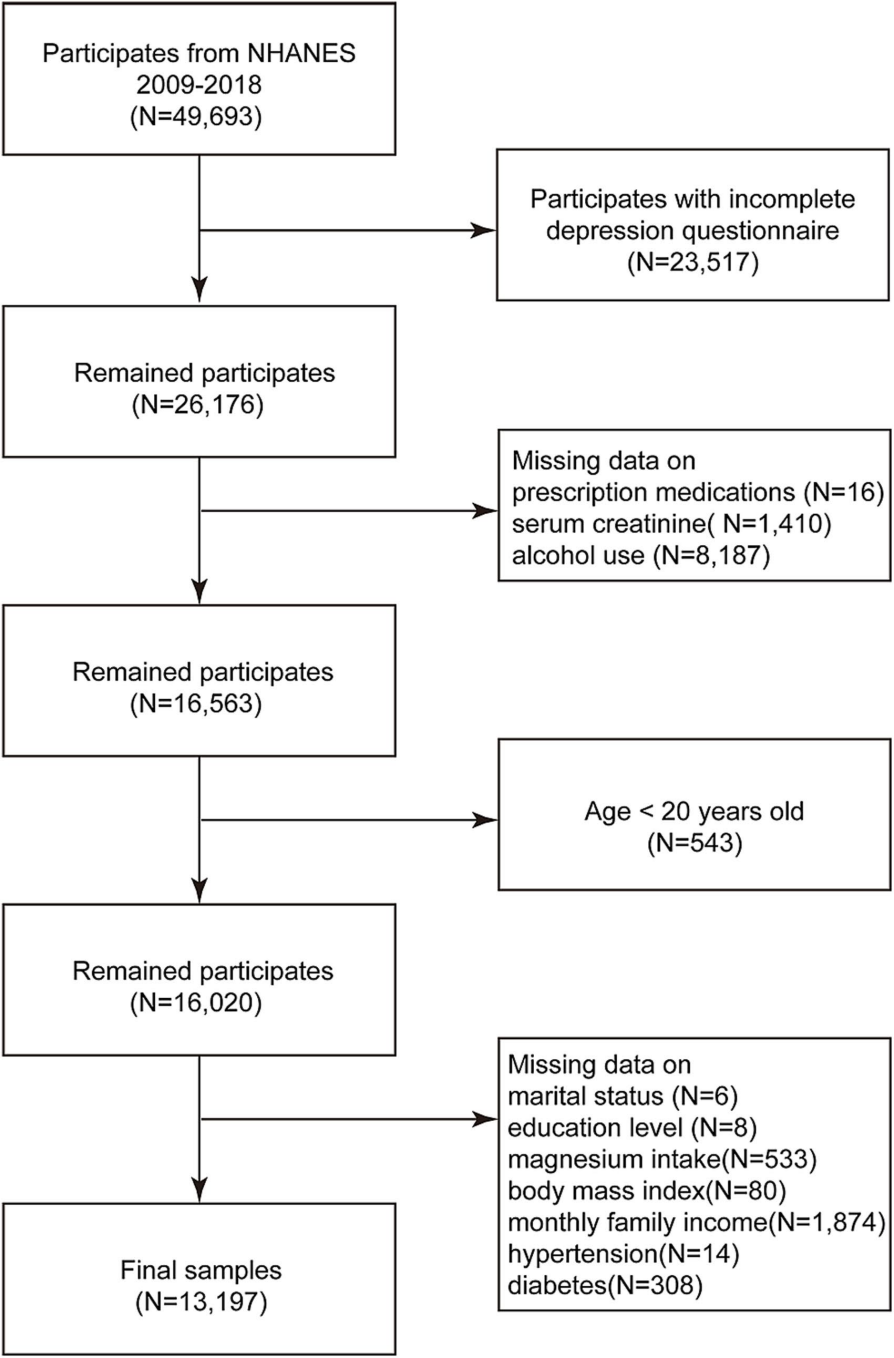
Flow chart for participants selection from the NHANES 2009–2018.

We incorporated the data of all participants from 5 cycles of the NHANES database spanning 2009 to 2018, comprising a total of 49,693 individuals. After rigorous screening, we excluded participants with missing depression status (*N* = 23,517), ambiguous prescription drug use status (*N* = 16), absent blood creatinine data (*N* = 1,410), unclear alcohol consumption status (*N* = 8,187), those younger than 20 years (*N* = 543), and those with missing covariates (*N* = 2,823). Ultimately, our analyses encompassed a final count of 13,197 participants, as depicted in Figure 1.

### 2.2 Calculation of MDS

The MDS was determined by quantifying four obtained criteria. Firstly, diuretic use was assessed, awarding one point for current use and zero points otherwise. Secondly, proton pump inhibitor use was considered, with one point awarded for current use and zero points for non-use. Thirdly, the estimated glomerular filtration rate (eGFR) was calculated using blood creatinine levels (22). Two points were given if the eGFR value was below 60, one point if it ranged from 60 to 90, and zero points otherwise. Lastly, alcohol abuse was evaluated, awarding one point if women consumed more than one drink per day or men drank more than two drinks per day, and zero points in other cases.

These calculations were based on the questionnaires administered to NHANES participants and their relevant physiological indicators. To facilitate in-depth analyses, apart from computing continuous MDS scores, we also categorized MDS into three groups as categorical variables: Low (0 points), Medium (1–2 points), and High (3 or more points).

### 2.3 Depression judgments

To assess the presence of depression among participants, we employed the Patient Health Questionnaire (PHQ-9) (23), a well-established and reliable tool for gauging the severity of depressive symptoms. This questionnaire comprises nine specific inquiries, each designed to evaluate the frequency of depressive symptoms, with scores ranging from 0 to 3. Specifically, answers indicating a lower frequency of symptoms receive a score of 0, while those reflecting a higher frequency are assigned a score of 3. By aggregating these individual scores, we determined the total score for each participant. Participants who attained a cumulative score of 10 or above across all nine questions were categorized as experiencing depression. The PHQ-9 scores were derived directly from the questionnaire administered to participants in the National Health and Nutrition Examination Survey.

### 2.4 Covariates

Drawing upon previous research (15, 24), we identified 10 potential confounding variables that could potentially influence the outcomes. These included factors such as gender, age, race, marital status, education level, monthly income, body mass index (BMI), hypertension status, diabetes status, and magnesium intake. These covariates encompassed four key domains: demographics, biochemistry, dietary factors, and physical measurements.

### 2.5 Statistical methods

We utilized R software (version 4.4.1) for the comprehensive data processing and statistical evaluation. In this study, categorical variables were presented as percentages and subjected to chi-square analysis for comparison. Meanwhile, continuous variables were expressed as the mean accompanied by the standard deviation (SD). Statistical significance was set at a two-sided *p*-value below 0.05.

To delve deeper into the correlation between MDS and depression, we employed multivariate logistic regression analysis, establishing three distinct models with varying degrees of covariate adjustments. Specifically, model 1 served as the baseline without any covariate adjustments, while model 2 accounted for gender, age, and ethnicity. Furthermore, model 3 built upon model 2 by incorporating additional factors such as marital status, education level, monthly income, BMI, hypertension, diabetes mellitus, and magnesium intake. Additionally, we conducted a subgroup analysis by grouping all the covariates and utilizing them as stratification factors. This approach aimed to capture the variability in the MDS-depression relationship across different population subsets.

## 3 Results

### 3.1 Comparison of baseline characteristics based on depression status

Overall, out of the 13,197 subjects participating in this study, 52.73% (6,959) were males. Among these participants, a substantial portion comprising 39.57% (5,222) were young adults aged 20–39 years. MDS across all subjects averaged 1.12 ± 0.90 (Mea*n* ± SD), and notably, 1,114 individuals (8.44%) exhibited depressive symptoms.

In a comparative analysis of depressed individuals versus those without depression, it was observed that the depressed group tended to have a higher proportion of females, were aged between 40 and 59, belonged to the Other Hispanic ethnic group, were divorced, had completed 9th-11th grade education, had a lower monthly income, were hypertensive, diabetic, overweight, consumed less magnesium, and had a higher MDS score. Table 1 offers a detailed breakdown of the baseline characteristics of the study participants, segmented based on the presence or absence of depressive symptoms.

**Table 1:** Characteristics of participants.

Characteristic	Overall	Non-depression	Depression	*P*-value
**Number**	13,197	12,083	1,114	
**Gender, *N* (%)**				<0.001
Male	6,959 (52.73%)	6,524 (53.99%)	435 (39.05%)	
Female	6,238 (47.27%)	5,559 (46.01%)	679 (60.95%)	
**Age, *N* (%)**				<0.001
Young adults (20–39)	5,222 (39.57%)	4,786 (39.61%)	436 (39.14%)	
Middle-aged adults (40–59)	4,514 (34.20%)	4,066 (33.65%)	448 (40.22%)	
Older adults (> = 60)	3,461 (26.23%)	3,231 (26.74%)	230 (20.65%)	
**Race, *N* (%)**				<0.001
Mexican American	1,771 (13.42%)	1,638 (13.56%)	133 (11.94%)	
Other Hispanic	1,256 (9.52%)	1,110 (9.19%)	146 (13.11%)	
Non-Hispanic White	6,043 (45.79%)	5,547 (45.91%)	496 (44.52%)	
Non-Hispanic Black	2,614 (19.81%)	2,379 (19.69%)	235 (21.10%)	
Other race	1,513 (11.46%)	1,409 (11.66%)	104 (9.34%)	
**Marital status, *N* (%)**				<0.001
Married	6,633 (50.26%)	6,280 (51.97%)	353 (31.69%)	
Widowed	657 (4.98%)	582 (4.82%)	75 (6.73%)	
Divorced	1,505 (11.40%)	1,297 (10.73%)	208 (18.67%)	
Separated	415 (3.14%)	327 (2.71%)	88 (7.90%)	
Never married	2,741 (20.77%)	2,472 (20.46%)	269 (24.15%)	
Living with partner	1,246 (9.44%)	1,125 (9.31%)	121 (10.86%)	
**Education level, *N* (%)**				<0.001
Less than 9th grade	744 (5.64%)	649 (5.37%)	95 (8.53%)	
9-11th grade	1,500 (11.37%)	1,303 (10.78%)	197 (17.68%)	
High school graduate	2,895 (21.94%)	2,615 (21.64%)	280 (25.13%)	
Some college or AA degree	4,392 (33.28%)	4,003 (33.13%)	389 (34.92%)	
College graduate or above	3,666 (27.78%)	3,513 (29.07%)	153 (13.73%)	
**Monthly family income, *N* (%)**				<0.001
$0 - $1,649	2,925 (22.16%)	2,514 (20.81%)	411 (36.89%)	
$1,650 - $4,599	5,204 (39.43%)	4,774 (39.51%)	430 (38.60%)	
$4,600 and over	5,068 (38.40%)	4,795 (39.68%)	273 (24.51%)	
**Hypertension, *N* (%)**				<0.001
Yes	4,237 (32.11%)	3,764 (31.15%)	473 (42.46%)	
No	8,960 (67.89%)	8,319 (68.85%)	641 (57.54%)	
**Diabetes, *N* (%)**				<0.001
Yes	1,397 (10.59%)	1,230 (10.18%)	167 (14.99%)	
No	11,800 (89.41%)	10,853 (89.82%)	947 (85.01%)	
**BMI, kg/m** ^**2**^ **, *N* (%)**				<0.001
Underweight (<18.5)	186 (1.41%)	165 (1.37%)	21 (1.89%)	
Normal weight (18.5–25)	3,669 (27.80%)	3,411 (28.23%)	258 (23.16%)	
Overweight (> = 25)	9,342 (70.79%)	8,507 (70.40%)	835 (74.96%)	
**Magnesium intake, mg, Mea*n* ± SD**	304.46 ± 138.05	307.40 ± 138.52	272.57 ± 128.69	<0.001
**MDS, Mean (±SD)**	1.12 ± 0.90	1.11 ± 0.89	1.27 ± 0.95	<0.001
**MDS categories, *N* (%)**				<0.001
Low (0)	3,139 (23.79%)	2,935 (24.29%)	204 (18.31%)	
Medium (1–2)	9,055 (68.61%)	8,273 (68.47%)	782 (70.20%)	
High (> = 3)	1,003 (7.60%)	875 (7.24%)	128 (11.49%)	

BMI, body mass index; MDS, magnesium depletion score; *N*, number; *P*-value, probability value; SD, standard deviation.

Continuous variables are expressed as mea*n* ± SD, with p-values derived from a linear regression analysis. Categorical variables, on the other hand, are reported as counts (%), and their corresponding *p*-values are obtained through a weighted chi-square test.

### 3.2 The association between MDS and depression

Table 2 displays the relationship between MDS and depression via a weighted multivariate logistic regression analysis. Across all three models, a statistically significant positive correlation was observed between MDS and depression (*p* < 0.05). Following the adjustment for all relevant variables, every incremental point in MDS corresponded to an 11% increase in the risk of developing depression (OR = 1.11, 95% CI 1.03–1.20). This positive relationship persisted when MDS was categorized into three distinct groups based on its score. In all three models, using the low-score subgroup as a benchmark, both the medium and high-score subgroups exhibited a greater likelihood of depression. In the comprehensive adjusted model, the medium-score subgroup showed a significantly increased likelihood of depression by 19%, when compared to the low-score subgroup (OR = 1.19, 95% CI 1.01–1.41). Similarly, the high-score subgroup exhibited a remarkably higher likelihood of depression, standing at 51% above the baseline (OR = 1.51, 95%CI 1.15–1.97).

**Table 2:** Association between MDS and depression.

Characteristic	Model1	Model2	Model3
	OR (95% CI) *p*-value	OR (95% CI) *p*-value	OR (95% CI) *p*-value
**MDS**	1.21 (1.13, 1.29) <0.0001	1.29 (1.20, 1.39) <0.0001	1.11 (1.03, 1.20) 0.0066
**MDS categories**			
Low (0)	1.0	1.0	1.0
Medium (1–2)	1.36 (1.16, 1.60) 0.0002	1.39 (1.18, 1.63) <0.0001	1.19 (1.01, 1.41) 0.0365
High (> = 3)	2.10 (1.67, 2.66) <0.0001	2.44 (1.89, 3.15) <0.0001	1.51 (1.15, 1.97) 0.0026

CI, confidence interval; MDS, magnesium depletion score; OR, odds ratio; *P*-value, probability value.

The relationship between MDS and depression was analyzed using multivariate logistic regression analysis.

Model 1: Covariates were not adjusted. Model 2: Adjusted for gender, age, and race as covariates. Model 3: Further adjusted for marital status, education level, household monthly income, BMI, hypertension, diabetes, and average magnesium intake based on Model 2.

### 3.3 Subgroup analyses

**Figure 2:**
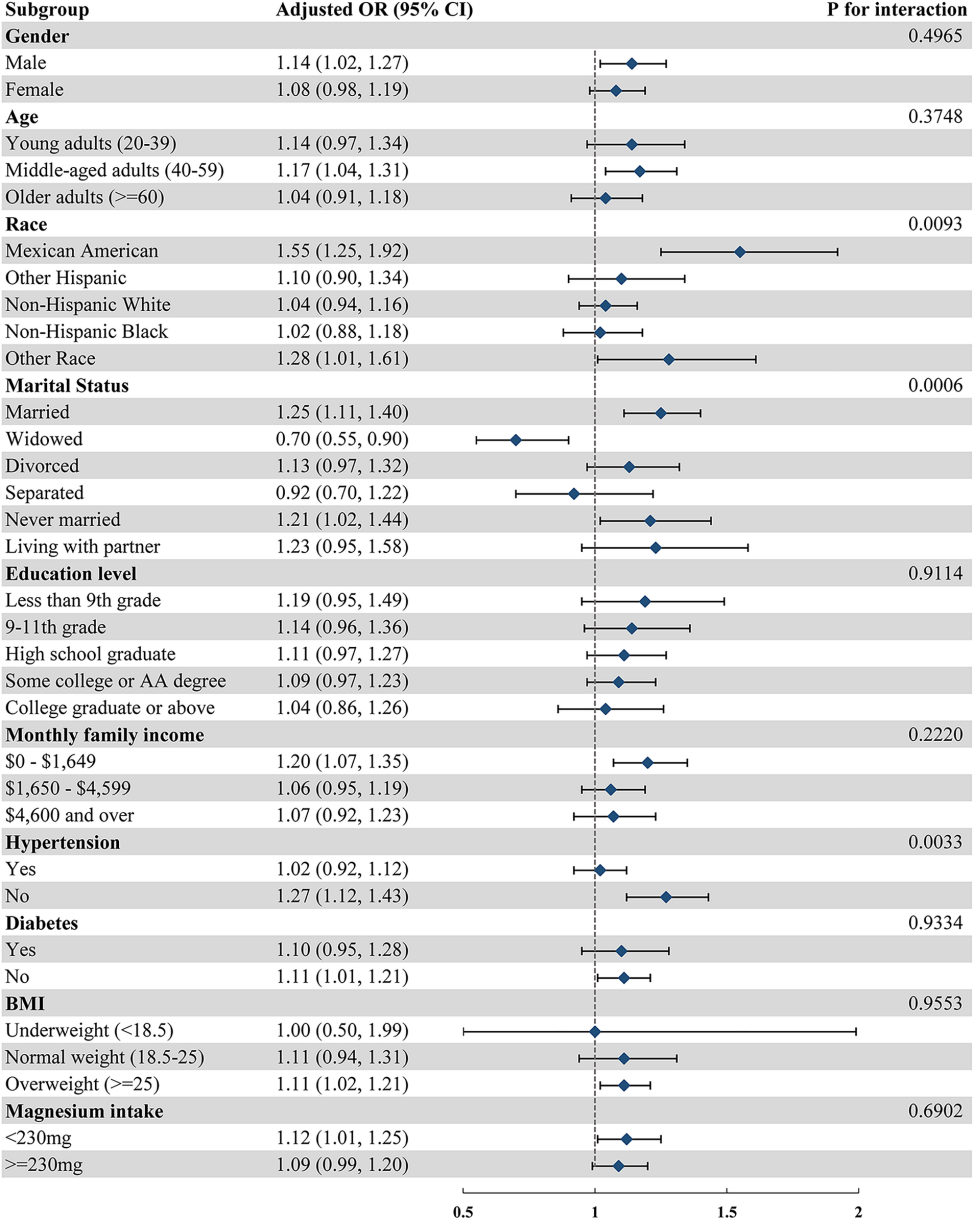
Forest plot of the association between MDS and depression across different subgroups.

To delve deeper into the varying associations between MDS and depression across various populations, we conducted subgroup analysis interaction tests. As Figure 2 illustrates, our findings reveal inconsistencies in these associations. Specifically, we found remarkable disparities in the linkage between MDS and depression when considering race, marital status, and hypertension, all with statistical significance (*p* < 0.05). The positive correlation between MDS and depression stood out among Mexican Americans, individuals of other races, married individuals, those who remain unmarried, and individuals free from hypertension. Nonetheless, it is noteworthy that a negative correlation emerged between MDS and depression in the widowed population (OR = 0.70, 95% CI 0.55–0.90). Interestingly, the consumption of magnesium did not seem to alter this relationship between MDS and depression.

## 4 Discussion

This cross-sectional study examined the relationship between MDS and depression, utilizing cohort data comprising 13,197 adult participants from the NHANES database spanning the years 2009 to 2018. Notably, our in-depth analysis uncovered a pronounced and statistically significant positive correlation between MDS and depression, consistent across the three models evaluated. Furthermore, this correlation exhibited noteworthy variations among subgroups stratified by marital status, race, and hypertension.

The relationship between magnesium and depression has garnered significant attention for quite some time. Magnesium plays a pivotal role in brain biochemistry, influencing numerous neurotransmission pathways linked to depression’s development (25). The research indicates that magnesium deficiency contributes to the pathophysiology of mood disorders (26). This may be because magnesium deficiency can affect glutamatergic transmission in the limbic system and cerebral cortex, brain regions that play crucial roles in the pathogenesis of depression (27). Furthermore, magnesium deficiency may alter the composition and signaling of N-methyl-D-aspartate receptors, leading to enhanced depressive-like behaviors (28). Additionally, magnesium exhibits certain immunoregulatory effects, which potentially interact with depression, particularly as magnesium deficiency may be associated with elevated levels of inflammatory markers such as serum C-reactive protein (29).

It is well-established in the literature that balancing magnesium levels in depressed patients has a beneficial impact on antidepressant effectiveness (30). Numerous studies have highlighted a substantial correlation between magnesium deficiency and depression (31, 32). Dietary magnesium intake may be associated with depression through its protective effects on the nervous system (33). A cross-sectional survey among Iranian postgraduate students noted a negative association between dietary magnesium intake and depression (34). Similarly, a cross-sectional study conducted in the Polish region among postmenopausal women revealed a negative correlation between serum magnesium levels and the severity of depressive symptoms (35).

In our current study, we employed MDS to assess the magnesium status of workers. MDS comprehensively accounts for the key factors that influence magnesium reabsorption in the human body, encompassing prescription drug use, renal function, and lifestyle habits. Regarding prescription drug use, studies have validated that prolonged use of diuretics and proton pump inhibitors can cause a decrease in magnesium levels in the body due to the interplay of various pathogenic mechanisms, potentially resulting in hypomagnesemia (36, 37). As for renal function, eGFR serves as a reliable indicator (38), and individuals with lower eGFR tend to have lower serum magnesium levels compared to those with normal renal function (39). Additionally, alcohol abuse disrupts intestinal magnesium absorption, a frequent cause of hypomagnesemia (40). In summary, the utilization of MDS offers a comprehensive assessment of magnesium status in humans.

Although the current study lacks a definitive biological mechanism to explain the interaction between MDS and depression, there are possible explanations for the underlying causes and mechanisms of this interaction. Notably, in subgroup analyses, the significant positive correlation between MDS and depression persisted among Mexican Americans (OR = 1.55, 95% CI 1.25–1.92) and individuals of Other Races (OR = 1.28, 95% CI 1.01–1.61). This suggests that the positive association between MDS and depression may not be uniform across different racial groups. Varied accessibility to mental health care (41) and disparities in renal magnesium handling (42) among different races could potentially influence the relationship between MDS and depression. Furthermore, alcohol consumption, a crucial factor in the calculation of MDS, exhibits distinct patterns across ethnic groups. For instance, Mexican-American adults have been identified as having high alcohol consumption rates within the Hispanic community (43), indicating that drinking habits may vary significantly between ethnicities.

In subgroup analyses, marital status emerged as a significant factor modulating the association between MDS and depression. Notably, the positive correlation between MDS and depression remained prominent in married (OR = 1.25, 95% CI 1.11–1.40) and never-married individuals (OR = 1.21, 95% CI 1.02–1.44). Conversely, among widowed individuals, this relationship shifted to a significant negative association (OR = 0.70, 95% CI 0.55–0.90). Previous research has highlighted the negative implications of marital dysfunction on health, particularly through depression (44). A study found a substantial link between the severity of depression and a lack of marital intimacy (45), indicating that such intimacy deficits can adversely affect individuals’ financial stability, behavior, and emotional well-being. A cross-sectional investigation among older adults in the United States revealed variations in energy expenditure among different marital statuses. Widowed individuals exhibited the lowest energy expenditure, rendering them susceptible to malnutrition (46). Furthermore, a robust association was observed between nutritional status, body magnesium levels, and depression. Moreover, numerous studies have documented that widows are more vulnerable to a range of conditions, including stroke, inflammation, and psychiatric disorders. This group also faces a significantly heightened risk of cardiovascular and all-cause mortality (47–49). These findings collectively suggest that such conditions may contribute to the observed association between MDS and depression.

In our subgroup analyses, we observed that hypertension modified the positive association between MDS and depression. Specifically, this association remained significant in the non-hypertensive population (OR = 1.27, 95% CI 1.12–1.43), but not among hypertensive patients (*p* < 0.05). The significance of magnesium in hypertension has been extensively researched. A meta-analysis revealed that magnesium intake exhibited the most beneficial effect on blood pressure reduction compared to other micronutrients (50). A study involving patients with essential hypertension highlighted a significant difference in magnesium excretion between hypertensive and non-hypertensive individuals (51), potentially explaining the variance in body magnesium levels between these two groups. Furthermore, several studies have demonstrated a high prevalence of comorbidities between hypertension and psychiatric disorders (52, 53). The physiological impacts of depression on cardiovascular health include hypothalamic–pituitary–adrenal axis and sympathoadrenal activation, rhythm disturbances, inflammation, and hypercoagulability. These factors could potentially contribute to the observed differences in the association between MDS and depression among hypertensive and non-hypertensive individuals.

In subgroup analysis, we observed an intriguing phenomenon: magnesium intake had no significant impact on the positive correlation between MDS and depression (p for interaction >0.05). This may be due to the complex absorption and consumption process of dietary magnesium within the human body, which could result in differences in functional magnesium status even among individuals with the same dietary magnesium intake (9). Furthermore, not all magnesium components are equally absorbed into the bloodstream (54).

To our knowledge, this marks the inaugural study to explore the correlation between MDS and depression. From a clinical perspective, our study provides evidence that magnesium deficiency may be a potential risk factor for depression, not only supporting previous research on the crucial role of magnesium in the nervous system and mental health but also offering new insights into the potential value of magnesium supplements as a preventive or adjuvant therapeutic means for depression. Among its strengths, this study boasts a sizable, nationally representative participant pool. We have accounted for confounding variables to bolster the reliability of our findings. Furthermore, through subgroup analyses, we have dissected the relationship between MDS and depression across diverse populations, thereby enhancing statistical rigor. However, our study does have its limitations. Firstly, the cross-sectional design precludes us from establishing a causal link between MDS and depression. Secondly, the extensive use of questionnaires in our data collection may have introduced deviations from the participants’ actual circumstances. Lastly, despite adjusting for a broad spectrum of 10 variables, we acknowledge that not all potential confounding factors have been accounted for.

## 5 Conclusion

In summary, there exists a notable positive correlation between MDS and depression. This finding underscores the importance of heightened vigilance regarding the risk of depression among individuals with elevated MDS levels. Reducing individual MDS scores could potentially play a pivotal role in mitigating the incidence of depression. To further validate our observations, additional prospective studies are warranted. In future research, we will continue to include covariates that may potentially influence the relationship between the two, and track changes in individual MDS to observe its impact on the incidence or severity of depressive symptoms, to establish causality.

## Data Availability

Publicly available datasets were analyzed in this study. This data can be found here: https://www.cdc.gov/nchs/nhanes/index.htm.
